# A case of crescentic glomerulonephritis in a patient with COVID-19 infection: A case report and literature review

**DOI:** 10.1097/MD.0000000000028754

**Published:** 2022-02-18

**Authors:** Mouhammad J. Alawad, Eihab A. Subahi, Haneen A. Al-Ani, Noheir M. Taha, Ijaz Kamal

**Affiliations:** a Department of Medical Education, Internal Medicine Residency Program, Hamad Medical Corporation, Doha, Qatar; b Department of Laboratory Medicine and Pathology, Hamad Medical Corporation, Doha, Qatar; c Department of Internal Medicine, Hamad Medical Corporation, Doha, Qatar.

**Keywords:** acute kidney injury (AKI), antineutrophilic cytoplasmic antibodies (ANCA), COVID-19 infection, focal segmental glomerulosclerosis (FSGS), glomerulonephritis (GN)

## Abstract

**Rationale:**

Kidney involvement with COVID-19 infection is a well-known complication, and the majority of kidney involvement is related to ischemic injury/acute tubular injury. However, there are some cases of glomerulonephritis, the etiology of which is not yet known, but an immune process is likely to be the trigger.

**Patient concerns:**

A 27-year-old man presented to our hospital with facial puffiness and lower-limb swelling.

**Diagnosis:**

Laboratory assessment revealed features of impaired kidney function with proteinuria and hematuria; COVID-19 polymerase chain reaction was positive, which was consistent with pauci-immune crescentic focal segmental glomerulonephritis.

**Intervention:**

After renal biopsy, the patient was started on methylprednisolone and rituximab. Due to worsening kidney parameters, he underwent intermittent hemodialysis as needed.

**Outcome:**

Kidney function tests partially improved; he was discharged on oral steroids with follow-up in the nephrology clinic to observe for the need for further hemodialysis.

**Lessons:**

We conducted a literature review of cases of glomerulonephritis associated with COVID-19 and described numerous types of glomerulonephritis. This report highlights the importance of recognizing emerging glomerulonephritis with COVID-19, the different pathological patterns of renal biopsies, and management interventions and responses.

## 1 Introduction

The COVID 19 pandemic has evolved and spread globally by 2020, affecting millions of people, with primary involvement of the respiratory system and a wide range of systemic involvements have been observed too.^[1]^ Kidney involvement is a well-established finding associated with COVID infection.^[^1^,^^2^^]^ Multiple mechanisms contribute to acute kidney injury, including infectious, immune-mediated, thrombotic, or toxic mechanisms.^[3]^ with only a few case reports of COVID 19 infection with glomerulonephritis^,^ ^[4]^ especially collapsing glomerulonephritis,^[^5^,^^6^^]^ a subtype of focal segmental glomerulosclerosis (FSGS), although other types of glomerulonephritis have been described.^[7]^ Here, we report a case of pauci-immune crescentic glomerulonephritis in a patient with COVID 19 infection that responded partially to steroids and rituximab. In a review of the literature, 24 cases of glomerulonephritis associated with COVID-19 were reviewed. Of these patients, 91% (22/24) underwent kidney biopsy, and collapsing FSGS was the most common type of glomerulonephritis (62.5%). Most patients were treated with immunomodulator therapy. The outcome was variable, with 45.8% of the patients remaining dialysis-dependent.

## 2 Case presentation

A 27-year-old male of Asian descent with no past medical history presented to the emergency department with facial puffiness and lower limb swelling. It started 5 days earlier and gradually worsened. He did not complain of fever, chills, cough, shortness of breath, or hemoptysis. It was not associated with a decrease in urine volume or change in urine color. He denied a recent history of pharyngitis, skin infection, or intake of nonsteroidal anti-inflammatory drugs. He had no family history of kidney or autoimmune diseases.

On examination, the patient had lower limb edema with puffiness around the eye. His blood pressure was 155/89 mm Hg, heart rate was 75 bpm, temperature was 36.9^o^, and oxygen saturation was 98%. His basic laboratory works up showed white blood cells count 10.2 × 10^9^/L, hemoglobin 8.1 g/dL, hematocrit 25.1%, mean corpuscle volume 90.9 femtoliter, platelets 184 × 10^9^/L. His blood urea level was elevated to 109.2 mg/dL, serum creatinine 6.06 mg/dL. His serum electrolyte, potassium, and bicarbonate levels were 142 mEq/L, raised serum potassium 6.2 mEq/L, bicarbonate 17.7 mEq/L. His C-reactive protein was 3.3 mg/L with a lactate dehydrogenase level of 363 U/L. The patient's urine protein ratio was high 665 mg/mmol. Urine analysis showed urine protein +3 and urine blood +3. Ultrasonography of the kidneys showed increased echogenicity, good corticomedullary differentiation, and trace perinephric free fluid.

A detailed work-up for acute kidney injury (AKI) was performed after consultation with the nephrology team. HIV Ag/Ab, HbsAg, HCV Ab, antinuclear antibodies, anti-GBM, and ANCA tests were all negative. His serum complement C4 level was normal. The serum complement C3 level was mildly low 0.87 a reference range (0.90 of 1.80). COVID-19 PCR was performed as a routine admission requirement and returned positive results. He was transferred to a COVID specialized facility where he was treated conservatively for COVID infection. He did not require antiviral drugs, such as remdesivir.

The patient underwent a kidney biopsy on the third day of admission, which was complicated by hematoma, and was treated conservatively. The patient underwent an emergency session of hemodialysis via the central line due to fluid overload and worsening renal parameters on the sixth day of admission, and was then placed on hemodialysis based on need.

**Figure 1:**
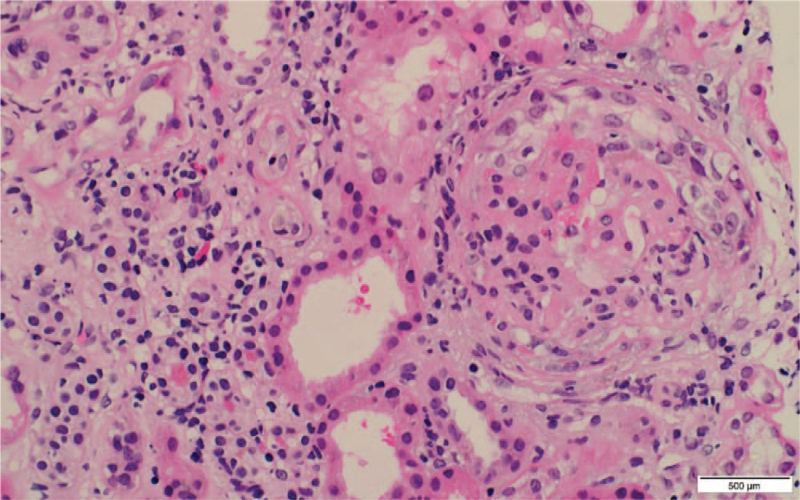
A glomerulus shows cellular crescent (involving nearly 50% of the glomerular circumference) with focal segmental glomerulosclerosis, fibrinoid necrosis, and karyorrhexis. H&E, ×40.

**Figure 2:**
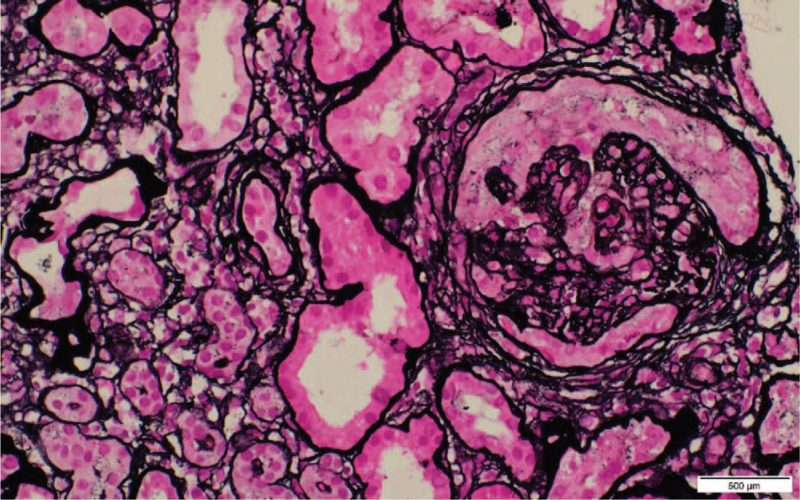
A circumferential cellular crescent in a glomerulus with segmental sclerosis. Silver stain, ×40.

**Figure 3:**
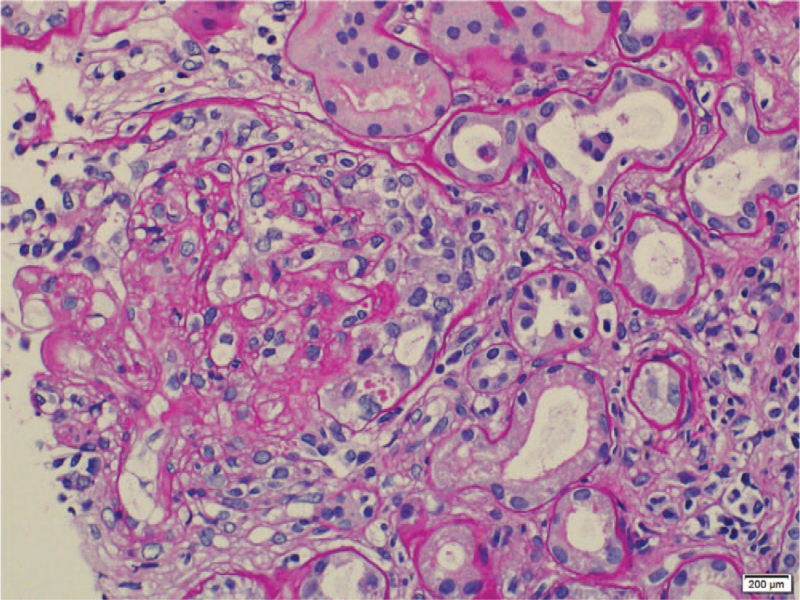
A circumferential cellular crescent (involving about 75% of the circumference) with focal fibrin deposition in the glomerular tuft (PAS stain, ×40).

**Figure 4:**
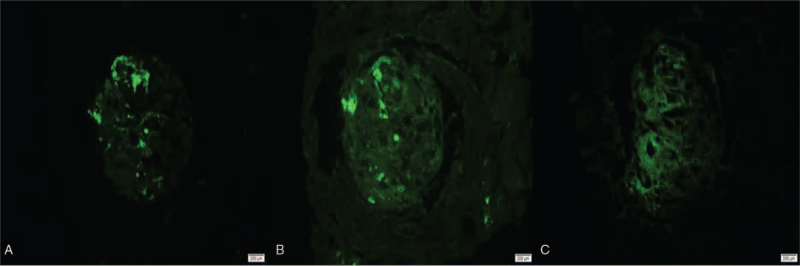
Indirect immunofluorescence showing (A) IgM shows lipohyaline deposit (1+), (B) IgA lipohyaline deposit (1+), (C) fibrinogen is negative.

Renal biopsy results were obtained on the eighth day, and no preliminary reports were provided. Biopsy revealed corticomedullary tissue with 6 glomeruli, 2 of which were globally sclerosed, and 2 showed segmental sclerosis with mesangial matrix expansion, mesangial cell proliferation, and focal fibrinoid necrosis with karyorrhexis and crescent formation. The other 2 viable glomeruli showed no significant abnormalities under light microscopy. Mild focal tubular and interstitial fubrosis (approximately 20–25% of the cortical tissue). There was a mild diffuse interstitial inflammatory infiltrate composed mainly of lymphocytes with occasional eosinophils. One medium-sized artery was thickened, with moderate subintimal fibrosis, medial hypertrophy, and focal mucoid changes. The arterioles were within normal limits. Indirect immunofluorescence microscopy of the biopsy specimen revealed mild, fine granular mesangial deposits with IgA and IgM (1+ for each). IgG, complement components C3 and C1q, and fibrinogen tests were negative. The biopsy report was diagnostic for pauci-immune focal and segmental glomerulonephritis with crescent formation. (Figs. 1, 2 and 3). We discussed the results with the pathologist, and IgA and IgM positivity was found in 60% of Pauci-immune cases. Although Pauci-immune crescentic glomerulonephritis with severe inflammation often shows positive findings for fibrinogen, in our case it was found to be negative, possibly due to the small biopsy volume, and only 2 glomeruli showed fibrinoid necrosis; repeated immunofluorescence showed the same findings—weak positivity of IgA and IgM, and negative for fibrinogen (Fig. 4).

The patient was treated with methylprednisolone 500 mg IV for 3 days and then received 1 dose of rituximab (1000 mg IV). His kidney function partially improved. Oral prednisone (60 mg) was administered once daily. The patient underwent intermittent hemodialysis as needed. After stabilization, the patient was discharged home on oral prednisone 60 mg once daily for months, with outpatient follow-up in the nephrology clinic to tapper down the stroids. The patient was scheduled to receive another dose of rituximab 2 weeks after the first dose. Upon discharge, his creatinine and urea levels were 3.1 mg/dL, urea 82.2 mg/dL.

## 3 Discussion

COVID-related kidney involvement is now a well-recognized manifestation of COVID-19.^[2]^ Multiple mechanisms, including cytotoxic injury due to direct viral invasion, acute tubular necrosis, thrombotic microangiopathy induced by a procoagulant state, and immune-mediated response derived from a cytokine storm.^[3]^ Probably immunological processes are probably the leading mechanisms for the development of glomerulonephritis. The characteristics of the reported cases of glomerulonephritis are shown in Table 1.^[^5^–^20^]^

**Table 1:** Characteristic of reported glomerulonephritis associated with COVID infection.

#	Author	Year	Age	Gender	Race	Co morbidities	Presentation	Serum Cr	Proteinuria	Hematuria	Imaging	Immunology	Biopsy	Treatment	Outcome
1	Uppal^[7]^	2020	64	Male	Black	Cryptogenic organizing pneumonia	Shortness of breath	7.87 mg/dL	Present	Present	Not available	p ANCA, ANA	pauci-immune crescentic glomerulonephritis	Glucocorticoids, rituximab	required HD, then improved
2	Uppal^[7]^	2020	46	Male	South Asian	Diabetes	Fever, diffuse purpuric rash	2.9 mg/dL	Present	Present	Not available	c ANCA	focal necrotizing glomerulonephritis	Glucocorticoids, rituximab	improved
3	Moeinzadeh^[10]^	2020	25	Male	Not available	NA	Rhinorrhea, arthralgia, pallor	3.7 mg/dL	Present	Present	Not available	c ANCA	crescentic glomerulonephritis	Glucocorticoids, cyclophosphamide, plasmapheresis	improved
4	Shah^[13]^	2020	8	Male	Not available	NA	Vomiting, diarrhea, facial swelling	0.32 mg/dL	Present	Present	Not available	–	not done	Glucocorticoids	improved
5	Alvarado^[14]^	2020	15	Male	Not available	NA	fever, generalized edema, and oliguria	0.55 mg/dL	Present	Not present	Not available	ANA, C4	not done	Glucocorticoids	improved
6	Pérez^[17]^	2021	88	Male	Not available	Hypertension, dyslipidemia	Dyspnea, edema	2.14 mg/dL	Present	Present	Not available	RF, C4	acute IgA Glomerulonephritis	Glucocorticoids	improved
7	Huang^[12]^	2020	65	Female	East Asian	IgA nephropathy, Hypertension	Dark-colored urine, flank pain	1.09 mg/dL	Present	Present	Unremarkable	–	acute IgA nephropathy	Glucocorticoid and angiotensin II receptor blocker	improved
8	Malhotra^[8]^	2020	64	Male	Black	Hypertension, DM, CKD III, HIV	Shortness of breath, fever	2.3 mg/dL	Present	Present	Not available	–	collapsing FSGS	Glucocorticoid	improved, HD dependent
9	Deshmukh^[19]^	2020	42	Male	South Asian	NA	Fever, cough, shortness of breath	1 mg/dL	Present	Present	Not available	–	collapsing FSGS	Angiotensin converting enzyme inhibitors	lost follow up
10	Noble^[20]^	2020	54	Male	Black	Hypertension, CKD II	Fever, cough, loss of smell	6.51 mg/dL	Present	Present	Unremarkable	–	collapsing FSGS	intravenous antibiotics, HD	HD dependent
11	Noble^[20]^	2020	45	Male	Black	DM, kidney transplant	Postural hypotension	4.67 mg/dL	Present	Present	Unremarkable	–	collapsing FSGS	Glucocorticoid, MMF, tacrolimus	HD dependent
12	Lazareth^[18]^	2020	29	Male	Black	Kidney transplant	Fever, cough, vomiting	6.04 mg/dL	Present	Not present	Not available	–	collapsing FSGS	Glucocorticoid, MMF, tacrolimus	improved
13	Kissling^[5]^	2020	63	Male	Black	Hypertension	Fever, shortness of breath	1.2 mg/dL	Present	Not present	Not available	–	collapsing FSGS	not available	improved
14	Larsen^[16]^	2020	44	Female	Black	Hypertension, DM, CKD	Fever, cough, vomiting, flank pain	4 mg/dL	Present	Present	Unremarkable	Anti SSA, ANA	collapsing FSGS	antibiotics, HD	HD dependent
15	Peleg^[15]^	2020	46	Male	Black	obesity, obstructive sleep apnea	Abdominal pain, nausea, decreased UOP	12.5 mg/dL	Present	Not present	↑ echogenicity	–	collapsing FSGS	Glucocorticoid	HD dependent
16	Basiratnia^[11]^	2021	17	Male	Not available	NA	Decrease in urine output, nausea, Vomiting	10.8 mg/dL	Present	Present	↑ echogenicity	–	acute necrotizing glomerulonephritis	Glucocorticoid	HD dependent
17	Basiratnia^[11]^	2021	16	Male	Not available	NA	fever, oliguria, dark urine	15.5 mg/dL	Present	Present	Not available	–	active necrotizing proliferative glomerulonephritis	Glucocorticoid	improved
18	Magoon^[9]^	2020	28	Female	Black	Asthma	Fever, cough, shortness of breath	6.5 mg/dL	Present	Not present	Unremarkable	–	collapsing FSGS	HD	improved
19	Magoon^[9]^	2020	56	Male	Black	HTN, CKD III	Fever, cough, vomiting	3.17 mg/dL	Present	Present	↑ echogenicity	–	collapsing FSGS	HD	improved
20–25	Wu^[6]^	2020	55	M: F 4:2	All black	–	Febrile illness	6.5 mg/dL	Present	2 present	–	–	collapsing FSGS		5 were HD dependent, 2 died

ANA = antinuclear antibodies, c ANCA = proteinase 3 antineutrophilic cytoplasmic antibodies, CKD = chronic kidney disease, Cr = creatinine, F = female, HD = hemodialysis, HTN = hypertension, M = male, NA = not applicable, p ANCA = myeloperoxidase antineutrophilic cytoplasmic antibodies, RF = rheumatoid factor, SSA = anti-Sjögren's syndrome-related antigen A autoantibodies.

Our patient presented with symptoms of fluid overload that developed over a week. Although he had no history of kidney disease and his renal function test results were within the normal range 9 months ago, we cannot exclude the possibility of an underlying kidney disease. The presentation of the reported cases varies from respiratory involvement to symptoms related to kidney injury, and some have very vague systemic symptoms. In our case, the patient was found to have nephrotic range proteinuria and microscopic hematuria, and all cases in Table 1 had proteinuria ranging from subnephrotic range to nephrotic with variable hematuria, which is related to different types of manifesting glomerulonephritis.

Renal biopsy was the most common type of GN in 91% of the total cases with collapsing glomerulonephritis (62.5%), a subtype of focal segmental glomerulosclerosis.^[^5^,^^6^^,^^8^^]^ Collapsing FSGS is more common in HIV-infected patients.^[^5^,^^9^^]^ The kidney biopsy of our patient showed crescentic glomerulonephritis, which was previously reported as a COVID-19 infection.^[10]^ Other types of glomerulonephritis-like necrotizing glomerulonephritis have also been reported.^[11]^ To note that case numbered 7 (Table 1) had IgA nephropathy before COVID infection.^[12]^ Some cases mentioned in the table (cases 4 and 5) did not undergo biopsy as they were young and presented with nephrotic syndrome symptoms. They were presumed to be minimal change diseases and responded well to glucocorticoids.^[^13^,^^14^^]^

The immunologic workup was positive in several cases.^[^7^,^^16^^,^^17^^]^ However, in our case, the workup was negative, including ANCA. Depending on this and renal biopsy results, he was labeled as ANCA-negative Pauci-Immune Crescentic Glomerulonephritis. Similar to all glomerulonephritis cases, our patient was treated with intravenous methylprednisolone for 3 days with 1 dose of rituximab. The patient required intermittent hemodialysis sessions because of worsening volume overload.

The prognosis in such cases varies from partial improvement in renal function to full dependence on regular dialysis. This may be related to the severity of acute kidney injury, underlying kidney disease, or other comorbidities. Our patient responded partially to the treatment and was discharged on oral prednisolone with a tapering regime and was scheduled to receive second dose of rituximab as an outpatient. However, his Cr level did not return to normal ranges, which can be attributed to a preexisting kidney disease. However, another possibility is that the patient had a post-biopsy hematoma and required multiple abdominal CT scans with contrast to follow-up the hematoma size. It is not yet clear whether the patient will undergo regular renal replacement therapy. Thus far, 46% of the reported cases have become dependent on hemodialysis.

## 4 Conclusion

Acute kidney injury can occur in COVID-related infections, and the most common cause is collapsing glomerulonephritis. However, crescentic glomerulonephritis is also associated with COVID-19, as in our case. We report this case with a literature review to highlight the different types of glomerulonephritis associated with COVID-19. It is also reasonable to perform renal biopsy in patients with suspected glomerular injuries. We hope that the publication of such data will give us more insight into the disease, risk factors, and interventions, such as medications, early or delayed renal replacement therapy, and prognosis.

## Acknowledgments

The authors acknowledge the internal medicine residency program for their motivation and support.

## Author contributions

Dr. Mouhammad J Alawad and Dr. Ijaz Kamal wrote and edited the manuscript; Dr. Eihab A Subahi and Dr. Haneen A Al-Ani were responsible for literature review. Dr. Noheir M. Taha provided us with the pathological images and interpretations. All authors approved the final manuscript.

**Conceptualization:** Mouhammad J Alawad.

**Data curation:** Eihab A. Subahi.

**Investigation:** Eihab A. Subahi, Haneen A Alani.

**Project administration:** Mouhammad J Alawad.

**Resources:** Haneen A Alani, Noheir M Taha.

**Supervision:** Mouhammad J Alawad, Ijaz Kamal.

**Visualization:** Noheir M Taha.

**Writing – original draft:** Mouhammad J Alawad.

**Writing – review & editing:** Ijaz Kamal.

## References

[1] Huang C Wang Y Li X. Clinical features of patients infected with 2019 novel coronavirus in Wuhan, China. Lancet 2020;395:497–506. 31986264 10.1016/S0140-6736(20)30183-5 PMC7159299

[2] Naicker S Yang CW Hwang SJ Liu BC Chen JH Jha V. The Novel Coronavirus 2019 epidemic and kidneys. Kidney Int 2020;97:824–8. 32204907 10.1016/j.kint.2020.03.001 PMC7133222

[3] Gagliardi I Patella G Michael A Serra R Provenzano M Andreucci M. COVID-19 and the kidney: from epidemiology to clinical practice. J Clin Med 2020;9:2506. 10.3390/jcm9082506 PMC7464116 32759645

[4] Sharma P Uppal NN Wanchoo R. COVID-19–associated kidney injury: a case series of kidney biopsy findings. J Am Soc Nephrol 2020;31:1948–58. 32660970 10.1681/ASN.2020050699 PMC7461689

[5] Kissling S Rotman S Gerber C. Collapsing glomerulopathy in a COVID-19 patient. Kidney Int 2020;98:228–31. 32471639 10.1016/j.kint.2020.04.006 PMC7156952

[6] Wu H Larsen CP Hernandez-Arroyo CF. AKI and collapsing glomerulopathy associated with COVID-19 and APOL1 high-risk genotype. J Am Soc Nephrol 2020;31:1688–95. 32561682 10.1681/ASN.2020050558 PMC7460910

[7] Uppal NN Kello N Shah HH. De Novo ANCA-associated vasculitis with glomerulonephritis in COVID-19. Kidney Int Rep 2020;5:2079–83. 32839744 10.1016/j.ekir.2020.08.012 PMC7439090

[8] Malhotra V Magoon S Troyer DA McCune TR. Collapsing focal segmental glomerulosclerosis and acute oxalate nephropathy in a patient with COVID-19: a double Whammy. J Investig Med High Impact Case Rep 2020;8:232470962096363. 10.1177/2324709620963635 PMC7543098 33019829

[9] Magoon S Bichu P Malhotra V. COVID-19–related glomerulopathy: a report of 2 cases of collapsing focal segmental glomerulosclerosis. Kidney Med 2020;2:488–92. 32775989 10.1016/j.xkme.2020.05.004 PMC7275990

[10] Moeinzadeh F Dezfouli M Naimi A Shahidi S Moradi H. Newly diagnosed glomerulonephritis during COVID-19 infection undergoing immunosuppression therapy, a case report. Iran J Kidney Dis 2020;14:239–42. 32361703

[11] Basiratnia M Derakhshan D Yeganeh BS Derakhshan A. Acute necrotizing glomerulonephritis associated with COVID-19 infection: report of two pediatric cases. Pediatr Nephrol 2021;36:1019–23. 33495896 10.1007/s00467-021-04944-w PMC7834948

[12] Huang Y Li X-J Li Y-Q. Clinical and pathological findings of SARS-CoV-2 infection and concurrent IgA nephropathy: a case report. BMC Nephrol 2020;21:504 doi:10.1186/s12882-020-02163-3. 33234164 10.1186/s12882-020-02163-3 PMC7685298

[13] Shah SA Carter HP. New-onset nephrotic syndrome in a child associated with COVID-19 infection. Front Pediatr 2020;08 471. 10.3389/fped.2020.00471 PMC7469478 32974243

[14] Alvarado A Franceschi G Resplandor E Sumba J Orta N. COVID-19 associated with onset nephrotic syndrome in a pediatric patient: coincidence or related conditions? Pediatr Nephrol 2020;36:205–7. 32852576 10.1007/s00467-020-04724-y PMC7450156

[15] Peleg Y Kudose S D’Agati V. Acute kidney injury due to collapsing glomerulopathy following COVID-19 infection. Kidney Int Rep 2020;5:940–5. 32346659 10.1016/j.ekir.2020.04.017 PMC7186120

[16] Larsen CP Bourne TD Wilson JD Saqqa O Sharshir MA. Collapsing glomerulopathy in a patient with COVID-19. Kidney Int Rep 2020;5:935–9. 32292867 10.1016/j.ekir.2020.04.002 PMC7142700

[17] Pérez A Torregrosa I D’Marco L. IgA-dominant infection-associated glomerulonephritis following SARS-CoV-2 infection. Viruses 2021;13:587. 33807151 10.3390/v13040587 PMC8066364

[18] Lazareth H Péré H Binois Y. COVID-19–related collapsing glomerulopathy in a kidney transplant recipient. Am J Kidney Dise 2020;76:590–4. 10.1053/j.ajkd.2020.06.009 PMC7354772 32668317

[19] Deshmukh S Zhou XJ Hiser W. Collapsing glomerulopathy in a patient of Indian descent in the setting of COVID-19. Ren Fail 2020;42:877–80. 32862747 10.1080/0886022X.2020.1811122 PMC7472468

[20] Noble R Tan MY McCulloch T. Collapsing glomerulopathy affecting native and transplant kidneys in individuals with COVID-19. Nephron 2020;144:589–94. 32894838 10.1159/000509938 PMC7573900

